# Metabolic Dysfunction-Associated Steatotic Liver Disease: An Update Narrative Review of the Therapeutic Potential of Combining Probiotics and Metformin

**DOI:** 10.3390/biomedicines14051147

**Published:** 2026-05-19

**Authors:** Syifa Mustika, Sri Utami, Nur Estu Wijayanti Saputri, Levrita Nindya Poetri, Putu Ijiya Danta Awatara, Achmad Rudijanto, Hery Djagat Purnomo, Cosmas Rinaldi A. Lesmana, Ahmad Taufiq

**Affiliations:** 1 Gastroenterohepatology Division, Department of Internal Medicine, Faculty of Medicine, Universitas Brawijaya, Malang 65111, Indonesia; 2 Department of Internal Medicine, Faculty of Medicine, Universitas Brawijaya, Malang 65145, Indonesia; sriutamidr22@gmail.com (S.U.); estusaputri@gmail.com (N.E.W.S.); levritalnp@gmail.com (L.N.P.); ijiyadanta19@gmail.com (P.I.D.A.); 3 Endocrine Metabolic and Diabetes Division, Department of Internal Medicine, Faculty of Medicine, Universitas Brawijaya, Malang 65111, Indonesia; achmad_rudijanto.fk@ub.ac.id; 4 Gastroenterohepatology Division, Department of Internal Medicine, Dr. Kariadi Hospital, Faculty of Medicine, Universitas Diponegoro, Semarang 50244, Indonesia; herydjagat@yahoo.co.id; 5 Hepatobiliary Division, Department of Internal Medicine, Faculty of Medicine, Universitas Indonesia, Depok 10430, Indonesia; medicaldr2001id@yahoo.com; 6 Department of Physics, Faculty of Mathematics and Natural Sciences, Universitas Negeri Malang, Malang 65145, Indonesia; ahmad.taufiq.fmipa@um.ac.id

**Keywords:** MASLD, MASH, probiotics, metformin, gut–liver axis, narrative review

## Abstract

Metabolic dysfunction-associated steatotic liver disease (MASLD) has replaced older exclusion-based terminology as the preferred term for steatotic liver disease associated with cardiometabolic risk factors. MASLD is now among the most common causes of chronic liver disease and may progress from simple steatosis to metabolic dysfunction-associated steatohepatitis (MASH), fibrosis, cirrhosis, and hepatocellular carcinoma. This updated rigorous narrative review synthesizes current evidence on MASLD diagnosis and management, with emphasis on the gut–liver axis and the therapeutic potential of combining probiotics with metformin. A structured narrative search was conducted in PubMed, PMC, ScienceDirect, Taylor & Francis, Cochrane Library, and Google Scholar using the keywords “MASLD”, “MAFLD”, “NAFLD”, “MASH”, “probiotics”, “synbiotics”, “metformin”, and “gut-liver axis”. The review was designed as a narrative synthesis rather than a systematic review. Current guidance supports stepwise risk stratification using serum fibrosis scores followed by elastography or advanced imaging when indicated. Ultrasonography remains accessible but has limited sensitivity for mild steatosis, is operator-dependent, and is not sufficient for comprehensive assessment of fibrosis or disease activity. Metformin is appropriate for type 2 diabetes mellitus and improves insulin resistance, but current guidelines do not recommend it as a targeted treatment for MASH because histological benefit has not been consistently demonstrated. Probiotics and synbiotics may improve aminotransferases, inflammatory markers, lipid parameters, intestinal barrier function, and gut dysbiosis; however, findings vary by strain, formulation, dose, treatment duration, population, and endpoint. The combination of probiotics and metformin is mechanistically plausible because it targets both metabolic dysfunction and intestinal dysbiosis, but human evidence remains limited. Larger, strain-specific, adequately powered trials using standardized MASLD criteria and clinically meaningful endpoints are required before routine clinical recommendations.

## 1. Introduction

Metabolic dysfunction-associated steatotic liver disease (MASLD) is currently recognized as one of the leading causes of chronic liver disease worldwide [1]. The disease is strongly associated with obesity, insulin resistance, type 2 diabetes mellitus (T2DM), dyslipidemia, and sedentary lifestyles. Despite its high global prevalence, public awareness remains limited [2]. MASLD represents a spectrum of liver abnormalities ranging from simple steatosis to metabolic dysfunction-associated steatohepatitis (MASH), which may progress to advanced fibrosis, cirrhosis, and hepatocellular carcinoma [3].

Recently, the International Liver Association proposed replacing the terminology nonalcoholic fatty liver disease (NAFLD) and metabolic dysfunction-associated fatty liver disease (MAFLD) with metabolic dysfunction-associated steatotic liver disease (MASLD) to reflect the metabolic pathophysiology better and reduce stigmatizing terminology associated with the previous nomenclature [4]. Simple steatosis is characterized by hepatic fat accumulation with minimal inflammation, whereas MASH involves hepatocyte ballooning, lobular inflammation, apoptosis, and varying degrees of fibrosis [5]. Increasing evidence supports the concept that the MASLD is clinically important because its burden is expanding in parallel with obesity, type 2 diabetes mellitus (T2DM), dyslipidemia, hypertension, and sedentary lifestyles [6]. According to the multisociety delphi consensus led by Rinella et al., MASLD is now considered the preferred terminology in contemporary hepatology practice [1]. In this review, the term MASLD is nowadays used, whereas MAFLD and NAFLD are mentioned when discussing previous studies or historical concepts.

The global prevalence of MASLD continues to increase in parallel with the rising burden of obesity and T2DM. Epidemiological studies estimate that approximately 25–35% of the global adult population is affected, making MASLD the most common chronic liver disease worldwide [7,8]. Progressive fibrosis remains the most important determinant of long-term morbidity and mortality, whereas cardiovascular disease represents the leading cause of death in affected individuals [9].

The pathogenesis of MASLD is multifactorial and involves complex interactions among insulin resistance, adipose tissue dysfunction, oxidative stress, inflammation, genetic susceptibility, and alterations in the gut microbiota [10]. Increasing evidence supports the central role of the gut–liver axis in disease progression. Gut dysbiosis may contribute to increased intestinal permeability, endotoxemia, hepatic inflammation, and fibrosis [11].

The increasing prevalence of MASLD, particularly in both developed and developing countries, needs effective strategies to prevent disease progression. Lifestyle interventions, including dietary modification and physical activity, remain the cornerstone of therapy but are often difficult to sustain over the long term. Consequently, adjunctive pharmacological and microbiota-targeted therapies are increasingly being explored. Metformin, a first-line antihyperglycemic agent, improves insulin sensitivity and glucose metabolism [12]. Meanwhile, probiotics may modulate gut microbiota composition, reduce inflammation, and improve intestinal barrier function [13]. Experimental and clinical studies suggest that probiotics may improve metabolic parameters and liver aminotransferase levels, particularly when combined with metformin [14,15]. Emerging evidence suggests that combined therapy may provide synergistic benefits by simultaneously modulating metabolic dysfunction and the gut–liver axis. Therefore, this narrative review aims to provide an update and a critical appraisal of the diagnosis and management of MASLD with particular emphasis on the therapeutic potential of combining probiotics and metformin.

## 2. Materials and Methods

This study was conducted as a narrative review focusing on the epidemiology, pathogenesis, diagnosis, and therapeutic management of MASLD, particularly the role of combining probiotics and metformin. Literature searches were performed using PubMed, PMC, ScienceDirect, Taylor & Francis, Cochrane Library, and Google Scholar databases. Articles published between 2013 and 2025 were searched using combinations of the following keywords: “MASLD”, “MAFLD”, “NAFLD”, “MASH”, “probiotics”, “synbiotics”, “metformin”, and “gut-liver axis”. Eligible sources included the following inclusion criteria: (1) articles written in English, (2) consensus statements, society guidelines, narrative reviews, systematic reviews, randomized controlled trials, observational studies, and mechanistic preclinical studies when they directly addressed MASLD or its predecessor NAFLD/MAFLD terminology, and (3) studies involving animal and human participants. Of the 512 articles initially identified, 32 studies met the inclusion criteria and were included in the final analysis.

## 3. MASLD at a Glance

### 3.1. Nomenclature and Epidemiology of MASLD

The term MASLD was introduced to replace the terminology Non-Alcoholic Fatty Liver Disease (NAFLD) following an international expert consensus aimed at aligning disease nomenclature with contemporary understanding of its underlying pathophysiology. The term NAFLD has been increasingly criticized because it was defined by the exclusion of significant alcohol consumption rather than by the presence of key pathogenic [16]. Accumulating evidence indicates that hepatic steatosis is strongly associated with metabolic dysfunction, including obesity, insulin resistance, type 2 diabetes mellitus, dyslipidemia, and hypertension, which collectively contribute to disease progression and adverse clinical outcomes [17]. In addition, the descriptor “non-alcoholic” was considered potentially stigmatizing and lacked diagnostic consistency due to variability in alcohol consumption thresholds among clinical guidelines [1]. Therefore, the adoption of MASLD emphasizes the central role of metabolic dysfunction in the development of steatotic liver disease and recognizes the condition as a hepatic manifestation of systemic metabolic dysregulation. This revised nomenclature is expected to improve disease characterization, facilitate risk stratification, and promote a more integrated approach to the management of both hepatic and cardiometabolic complications.

The global prevalence of MASLD has increased substantially over the past decades and is estimated to affect approximately 25–35% of the adult population worldwide, representing nearly one billion individuals [18]. Regional variations are observed, with relatively lower prevalence reported in parts of Africa and higher rates in the Middle East, South America, and certain regions of Asia [6,18]. In the United States, MASLD prevalence continues to rise in parallel with increasing obesity and type 2 diabetes mellitus (T2DM), with projections suggesting a continued upward trend through 2030 and impacting more than 30% of people worldwide [19]. Importantly, a considerable proportion of patients with T2DM and normal liver enzyme levels may still have underlying steatohepatitis or fibrosis, indicating that aminotransferase levels are not always reliable indicators of disease severity [6,20]. Fibrosis stage and metabolic dysfunction-associated steatohepatitis (MASH)—previously termed non-alcoholic steatohepatitis (NASH)—are key severity factors in patients with MASLD, as progression to more advanced stages is linked to greater risks of overall mortality and liver-related complications, including hepatocellular carcinoma and hepatic decompensation [21]. MASH is characterized not only by hepatic lipid accumulation but also by hepatic inflammation and hepatocellular injury [17].

### 3.2. Pathophysiology of MASLD

The pathogenesis of MASLD is complex and multifactorial, involving insulin resistance, oxidative stress, lipotoxicity, genetic predisposition, environmental influences, and alterations in the gut microbiota [22,23]. Rather than the earlier “two-hit” hypothesis, MASLD progression is better explained by the multiple-parallel hit hypothesis, in which insulin resistance and metabolic syndrome, adipose tissue inflammation, mitochondrial dysfunction, oxidative stress, and gut–liver axis disturbances act simultaneously [24]. Insulin resistance promotes increased de novo hepatic lipogenesis and enhanced lipolysis in adipose tissue, leading to excessive free fatty acid influx into the liver [25]. This process results in intrahepatic lipid accumulation, mitochondrial dysfunction, endoplasmic reticulum stress, and excessive production of reactive oxygen species. Overall, MASLD represents a metabolism-driven liver disease characterized by complex interactions among insulin resistance, adipose tissue dysfunction, oxidative stress, and gut microbiota alterations. Its increasing global prevalence and strong association with cardiometabolic comorbidities underscore the need for early detection and targeted therapeutic strategies.

### 3.3. Clinical Diagnosis and Risk Stratification of MASLD

The diagnosis of MASLD, especially during the early stages of disease progression, is frequently considered a “silent” condition because it commonly remains asymptomatic. Nevertheless, accumulating evidence highlights the importance of prompt detection and appropriate management to reduce the risk of progression toward serious hepatic and metabolic complications. Screening for MASLD is particularly important in patients with metabolic risk factors, including obesity, type 2 diabetes, hypertension, dyslipidemia, and metabolic syndrome. Among these, individuals with type 2 diabetes are at especially high risk, with a prevalence reaching up to 65% in this population [17].

Initial screening should include liver function tests (LFTs) and the calculation of the serum-based non-invasive fibrosis scores, such as FIB-4 (Fibrosis-4 Index), a widely validated and simple tool for assessing fibrosis risk [17,26]. In some cases, alternative scores such as the NAFLD Fibrosis Score (NFS) or the Enhanced Liver Fibrosis (ELF) test may provide additional value, because it is based on age, aminotransferases (AST/ALT), platelet count, BMI, and metabolic parameters. These scores have been validated in large cohorts for identifying advanced fibrosis and are recommended as first-line screening tools in primary care, particularly for intermediate-risk patients or when a higher accuracy in detecting advanced fibrosis is required [27,28].

Imaging modalities are also essential in MAFLD diagnosis. Ultrasonography (US) is widely used due to its accessibility and cost-effectiveness for detecting hepatic steatosis, but it offers limited utility for fibrosis assessment. Conventional ultrasound has reduced sensitivity for mild steatosis, is operator-dependent, performs less well in severe obesity, and cannot reliably quantify steatosis or stage fibrosis. It is therefore not the preferred modality for comprehensive disease assessment when more accurate tools are available [1,29,30]. More advanced imaging techniques provide greater diagnostic accuracy. Magnetic Resonance Imaging–Proton Density Fat Fraction (MRI-PDFF) is currently considered the most accurate non-invasive modality for quantifying hepatic steatosis [31]. For fibrosis assessment, Magnetic Resonance Elastography (MRE) has demonstrated superior diagnostic performance compared with other non-invasive modalities. Therefore, Vibration-Controlled Transient Elastography (VCTE), commercially known as FibroScan^®^, is preferred for staging fibrosis in MAFLD. It measures liver stiffness via shear wave velocity and is recommended in international practice guidelines for fibrosis risk stratification [27,28].

In select cases, liver biopsy is the gold standard for diagnosing hepatic steatosis, steatohepatitis, fibrosis, and cirrhosis [22]. Liver biopsy is indicated when non-invasive tests yield conflicting results or if there is clinical suspicion of advanced fibrosis, cirrhosis, or overlapping liver diseases. Nevertheless, liver biopsy is an invasive procedure associated with several limitations and procedural risks, including post-biopsy pain occurring in up to 50% of patients and serious complications such as hemorrhage, which may occur in approximately 0.6–1% of cases [26]. However, biopsy is invasive, costly, associated with sampling variability, and carries procedural risks. Therefore, it is reserved for selected patients with suspected advanced fibrosis or diagnostic uncertainty.

In the diagnostic work-up for MASLD, additional tests are necessary to exclude other liver conditions that may coexist or mimic MASLD, including hepatitis B and C panels, autoimmune liver disease markers (ANA and ASMA), and iron studies to rule out hereditary hemochromatosis. Current international guidelines, including the European Association for the Study of the Liver (EASL), recommend a stepwise algorithm combining serum biomarkers and imaging techniques to identify patients at risk of advanced fibrosis [27]. Balanced overview of diagnostic modalities and limitations in MASLD, as shown in Table 1.

### 3.4. Current Management in Brief of MASLD

Management of treating MASLD as a multisystem metabolic disease. Sustained weight loss remains the cornerstone of therapy. Weight reduction of approximately 5% can improve steatosis, while greater weight loss, often 7–10% or more, is associated with improvement in steatohepatitis and may improve fibrosis in some patients [1,29,32,33]. Dietary patterns emphasizing calorie reduction, Mediterranean-style foods, reduced saturated fat, limited refined carbohydrates, and avoidance of sugar-sweetened beverages are generally recommended. Physical activity improves hepatic and cardiometabolic outcomes even when weight loss is modest [29,32].

Management must also address T2DM, dyslipidemia, hypertension, obesity, sleep apnea, CKD, and cardiovascular risk. Pharmacotherapy is selected according to comorbidities and fibrosis stage. Some metabolic agents improve weight or glycemic control and may reduce liver fat, but evidence for histological benefit varies. Importantly, metformin should not be framed as a specific MASH therapy despite its value for T2DM and insulin resistance [12,29]. Microbiota-targeted interventions, including probiotics, synbiotics, and fecal microbiota transplantation, remain investigational adjuncts rather than standard-of-care treatments [15,34,35].

## 4. Gut–Liver Axis: Interplay of Probiotics and Metformin in MASLD

### 4.1. Role of Gut Dysbiosis in MASLD Progression

Intestinal dysbiosis plays a critical role in MASLD pathogenesis. Increased intestinal permeability facilitates the translocation of bacterial endotoxins, such as lipopolysaccharide (LPS), thereby activating Kupffer cells and hepatic stellate cells and promoting hepatic inflammation and fibrogenesis [23,36]. Alterations in gut microbiota composition, including increased Verrucomicrobia, Bacteroidetes, Proteobacteria, and Cyanobacteria, have been associated with increased intestinal permeability, elevated circulating LPS levels, and enhanced production of pro-inflammatory cytokines such as tumor necrosis factor-alpha (TNF-α) and interleukin-6 (IL-6) [17]. These inflammatory mediators activate several intracellular signaling pathways, including Toll-like receptor 4/nuclear factor-kappa B (TLR4/NF-κB) and AMP-activated protein kinase (AMPK), thereby promoting hepatic lipid accumulation, inflammation, and insulin resistance [7]. Mechanistic pathways linking dysbiosis to MASLD include dysregulated short-chain fatty acid production, altered bile acid metabolism, endogenous ethanol production, impaired choline metabolism, and activation of pro-inflammatory cytokines such as tumor necrosis factor (TNF)-α and interleukin-6 [36].

Increased LPS exposure also promotes polarization of Kupffer cells toward the pro-inflammatory M1 phenotype, resulting in enhanced secretion of TNF-α, IL-1β, IL-12, and reactive oxygen species (ROS), which collectively intensify hepatic inflammation and oxidative stress [37]. Intestinal dysbiosis additionally disrupts gut barrier integrity through upregulation of serine-threonine kinase 39 (STK39), facilitating translocation of LPS into the systemic circulation and amplifying inflammatory responses [38]. Furthermore, downstream cytokine signaling pathways contribute to sustained hepatic injury, as TNF-α activates NF-κB signaling while IL-6 induces the Janus kinase/signal transducer and activator of transcription 3 (JAK/STAT3) pathway, promoting transcription of genes involved in chronic inflammation and metabolic dysfunction [39]. Collectively, these findings underscore the central role of the gut–liver axis, microbiota dysbiosis, and LPS-mediated inflammatory signaling in the development and progression of MASLD.

### 4.2. Gut–Liver Axis as a Therapeutic Target

The gut–liver axis provides a biologically plausible target for adjunctive MASLD therapy. The portal circulation exposes the liver to microbial products, bile acids, short-chain fatty acids, and diet-derived metabolites. When intestinal barrier integrity is disrupted, microbial translocation can promote hepatic inflammation through toll-like receptor activation, oxidative stress, and hepatic stellate cell activation. Dysbiosis may also alter bile acid pools, farnesoid X receptor signaling, glucagon-like peptide-1 secretion, and choline metabolism, thereby linking intestinal microbial ecology to hepatic lipid accumulation and insulin resistance [23,36,40,41,42].

Probiotics are defined as live microorganisms that confer health benefits when administered in adequate amounts. In MASLD, proposed mechanisms include restoration of microbial diversity, competitive inhibition of pathogenic bacteria, improved tight-junction integrity, reduced endotoxemia, lower inflammatory cytokine production, altered bile acid metabolism, and improved oxidative stress responses [35,43,44]. Synbiotics combine probiotics with prebiotics and may enhance colonization or metabolic effects, although trial results remain heterogeneous [34,35,45,46].

### 4.3. Probiotic Evidence: Benefits and Heterogeneity

Clinical trials of probiotics and synbiotics in NAFLD/MAFLD/MASLD have generally evaluated surrogate endpoints such as ALT, AST, gamma-glutamyl transferase, lipid profile, insulin resistance, inflammatory markers, ultrasound grade, fatty liver index, CAP, or liver stiffness. Several trials and meta-analyses report improvements in liver enzymes, inflammatory markers, lipid parameters, body mass index, and insulin sensitivity [34,35,43,45,47,48]. However, these effects are not uniform. Some trials report microbiome changes without significant improvement in liver fat or fibrosis, and others show no clear clinical benefit beyond diet and lifestyle intervention [46,49,50].

The main interpretive challenge is heterogeneity. Probiotic genera are often reported only at the genus level, but clinical effects are strain-specific. Trials vary in the use of Lactobacillus, Bifidobacterium, Streptococcus, Bacillus, Lactococcus, Propionibacterium, multi-strain mixtures, yogurt-based interventions, and synbiotic formulations. Duration ranges from weeks to more than one year, sample sizes are often small, baseline fibrosis severity differs, and endpoints are inconsistent [35]. As a result, probiotics may be considered promising adjuncts, but they cannot yet be recommended as standardized disease-modifying therapy for all patients with MASLD.

### 4.4. Metformin: Mechanistic Rationale and Guideline Limitation

Metformin is a first-line antihyperglycemic agent for T2DM and has long been evaluated in fatty liver disease because insulin resistance is central to pathogenesis. Metformin decreases hepatic gluconeogenesis, improves peripheral insulin sensitivity, activates AMP-activated protein kinase, reduces lipogenesis, and may influence gut microbiota composition and intestinal bile acid signaling [12,51,52]. These effects are highly relevant to patients with MASLD and T2DM.

However, clinical evidence has not consistently demonstrated histological improvement in MASH or fibrosis. Current guidelines therefore do not recommend metformin specifically for MASH treatment, while still supporting its use for appropriate glycemic indications in patients with MASLD and T2DM [12,29]. This distinction is essential: metformin may improve metabolic risk and possibly aminotransferases in some patients, but it should not be represented as an established anti-MASH therapy.

## 5. Potential Role of Combination Metformin and Probiotics in MASLD

The rationale for combining metformin and probiotics is based on complementary and potentially synergistic effects. Metformin targets insulin resistance, hepatic glucose production, AMPK signaling, and cardiometabolic risk. Probiotics target dysbiosis, intestinal barrier dysfunction, endotoxin translocation, bile acid signaling, and inflammatory responses. Together, they may reduce the metabolic and microbial inputs that drive hepatic steatosis and inflammation. This concept is particularly relevant for MASLD patients with T2DM, obesity, or evidence of gut dysbiosis [12,14,23,34,35,36,40,41,45,51,52].

The direct human evidence for combination therapy remains limited. Shavakhi et al. randomized 64 adults with biopsy-confirmed NASH to metformin plus probiotic or metformin plus placebo for 6 months [14]. The combination group showed greater reductions in ALT and AST and improved ultrasound grading compared with metformin alone [30]. This study is important because it directly tested combination therapy, but it also has major limitations: it used older NASH terminology, had a small sample size, relied on aminotransferases and ultrasound rather than repeat histology or MRI-PDFF/MRE, did not define modern MASLD subgroups, and did not provide strain-specific microbiome response data.

Preclinical evidence also supports biological plausibility but cannot be directly extrapolated to MASLD. Patel et al. evaluated metformin and a multi-strain probiotic preparation in ethanol-induced hepatic injury models and reported reduced oxidative stress, endoplasmic reticulum stress, lipid accumulation, inflammatory signaling, and histopathological injury with combined treatment [53]. Although the model was alcohol-related rather than metabolic steatotic liver disease, it reinforces the concept that metformin–probiotic combinations may affect oxidative, inflammatory, and lipid pathways shared across liver injury phenotypes.

Emerging preclinical work continues to highlight metabolic signaling pathways that may be relevant to future adjunctive strategies. For example, Yuenyong et al. reported that tangeretin improved high-fat diet/fructose-induced metabolic dysfunction and fatty liver in rats by reducing oxidative stress, inflammation, and enhancing IRS/Akt signaling, with metformin used as a comparator [48]. Such data do not support immediate clinical use of tangeretin in MASLD, but they illustrate the mechanistic emphasis on insulin signaling, oxidative stress, and inflammation that should guide future combination. Structured evidence map of metformin–probiotic combination and selected probiotic studies relevant to MASLD, as shown in Table 2.

Taken together, the evidence supports a hypothesis rather than a standard treatment recommendation. Combination therapy may be most rational in patients with MASLD and T2DM, patients with insulin resistance and dysbiosis, or patients in whom metformin is already clinically indicated. However, probiotics should not be used as a substitute for weight reduction, glycemic control, lipid management, blood pressure control, or fibrosis risk stratification.

Future studies should use the current MASLD/MASH nomenclature, clearly specify probiotic strain and dose, confirm viability, stratify participants by T2DM, obesity, baseline fibrosis, and microbiome phenotype, and include clinically meaningful endpoints. Preferred endpoints include MRI-PDFF for steatosis, VCTE or MRE for fibrosis risk, validated serum biomarkers, cardiometabolic outcomes, quality of life, adverse events, adherence, and histology. Trials should also compare metformin alone, probiotics alone, combined therapy, and placebo or lifestyle control to determine whether any benefit is additive or synergistic.

## 6. Strengths and Limitations

This revised review has several strengths. First, it updates the terminology from MAFLD to MASLD and aligns the discussion with the 2023 multisociety nomenclature consensus and recent society guidance [1,29]. Second, it reorganizes the manuscript around a clear narrative aim: current MASLD diagnosis and management with focused appraisal of probiotics, metformin, and their potential combination. Third, it provides a more balanced diagnostic section that recognizes the limitations of ultrasound and serum biomarkers, including the need for caution in patients with CKD or other comorbid conditions. Fourth, it adds structured evidence tables to improve transparency and readability.

The review also has limitations. It is a narrative review and does not include PRISMA-based study selection, formal risk-of-bias scoring, or meta-analysis. Much of the clinical probiotic literature was conducted under NAFLD or MAFLD terminology, and the applicability to current MASLD criteria requires careful interpretation. Direct human evidence for combined metformin and probiotics is limited, and existing studies often rely on surrogate outcomes rather than histological or MRI-based endpoints. Probiotic heterogeneity also limits generalizability because effects may depend on strain, dose, formulation, viability, treatment duration, diet, baseline microbiome, and host metabolic phenotype.

## 7. Conclusions

MASLD is a common and clinically consequential metabolic liver disease requiring early identification, fibrosis risk stratification, and integrated cardiometabolic management. The revised nomenclature should be applied consistently, with MAFLD and NAFLD terminology used only when discussing historical literature. Diagnostic evaluation should not rely solely on aminotransferases or conventional ultrasound; a stepwise approach using serum fibrosis scores and elastography, with MRI-based tools for selected cases, is more consistent with current guidance. Metformin remains important for T2DM management but is not recommended as a specific therapy for MASH. Probiotics and synbiotics show potential as adjunctive therapies through gut–liver axis modulation, but trial heterogeneity and inconsistent endpoints limit definitive conclusions. The combination of metformin and probiotics is mechanistically appealing and supported by limited clinical and preclinical evidence, but it remains investigational. Well-designed, adequately powered, strain-specific randomized trials using current MASLD criteria and robust liver and metabolic endpoints are needed before routine recommendations.

## Figures and Tables

**Table 1:** Balanced overview of diagnostic modalities and limitations in MASLD.

Modality	Main Role	Strengths	Limitations and Clinical Caveats
Liver biopsy	Reference gold standard for MASH diagnosis and fibrosis staging.	Direct histological assessment of steatosis, ballooning, inflammation, and fibrosis.	Invasive, costly, sampling variability, not suitable for routine screening; reserved for diagnostic uncertainty or suspected advanced disease.
FIB-4/NAFLD Fibrosis Score	First-line fibrosis risk stratification in primary care and metabolic clinics.	Low cost, uses routine laboratory data, high negative predictive value at low-risk thresholds.	Indeterminate zone common; affected by age, AST/ALT variability, platelet disorders, acute illness, viral hepatitis, alcohol exposure, and CKD-related comorbidity.
Conventional ultrasonography	Initial detection of moderate-to-severe steatosis when resources are limited.	Accessible, inexpensive, non-invasive.	Limited sensitivity for mild steatosis, operator-dependent, less reliable in obesity, cannot stage fibrosis or quantify dynamic change precisely.
VCTE with CAP	Second-line assessment of liver stiffness and steatosis.	Point-of-care, repeatable, guideline-supported for fibrosis risk stratification.	Performance affected by obesity, inflammation, congestion, fasting status, probe selection, and high failure or unreliable measurement rates in some patients.
MRI-PDFF	Quantitative liver fat measurement in trials or complex cases.	High accuracy and reproducibility for steatosis quantification.	Cost, availability, and limited routine access.
MRE	Advanced non-invasive fibrosis assessment.	Excellent diagnostic performance for fibrosis and is useful when other tests are discordant.	Cost, availability, and contraindications to MRI.
Inflammatory/coagulation biomarkers	Research or adjunctive assessment in selected settings.	Local studies have explored AST plus TNF-alpha and PAI-1 as markers linked to steatohepatitis or fibrosis [24,25].	Not yet standardized for routine MASLD risk stratification; requires external validation and context-specific interpretation.

**Table 2:** Structured evidence map of metformin–probiotic combination and selected probiotic studies relevant to MASLD/MASH.

Study/Source	Design and Population	Intervention	Duration	Main Outcomes	Critical Interpretation
Shavakhi et al., 2013 [14]	Double-blind randomized clinical trial; 64 adults with biopsy-confirmed NASH.	Metformin plus probiotic (Protexin) versus metformin plus placebo.	6 months	Combination therapy reduced ALT and AST and improved ultrasound steatosis grading more than metformin alone.	Only direct human combination trial identified; small sample; older NASH criteria; no repeat histology, VCTE, MRI-PDFF, or microbiome profiling.
Patel et al., 2021 [53]	In vitro HepG2 and RAW 264.7 cells plus in vivo ethanol-induced hepatic injury model.	Metformin plus multi-strain probiotic preparation.	Preclinical protocol	Reduced cellular injury, oxidative stress, ER stress, inflammatory response, lipid accumulation, and histopathological injury.	Mechanistically useful, but ethanol injury is not MASLD; human metabolic disease translation remains uncertain.
Malaguarnera et al., 2012 [48]	Clinical trial in NASH.	Bifidobacterium longum plus fructo-oligosaccharides with lifestyle modification.	24 weeks	Improved insulin resistance, TNF-alpha, CRP, AST, endotoxin, steatosis, and NASH activity index.	Supports gut–liver axis targeting but not metformin combination; formulation-specific effects.
Sepideh et al., 2016 [54]	Double-blind randomized clinical trial in NAFLD.	Multi-strain Lactobacillus, Bifidobacterium, and Streptococcus probiotic.	8 weeks	Improved glycemic and inflammatory indices.	Short duration and surrogate outcomes; long-term fibrosis effects unknown.
Kobyliak et al., 2018 [55]	Randomized clinical trial in NAFLD.	Multi-strain probiotic including Bifidobacterium, Lactobacillus, Lactococcus, and Propionibacterium.	8 weeks	Reduced fatty liver index, cytokines, and aminotransferases.	Promising but short-term and dependent on composite/surrogate outcomes.
Duseja et al., 2019 [56]	Randomized double-blind proof-of-concept study in biopsy-confirmed NAFLD.	High-potency multi-strain probiotic.	1 year	Improved NAFLD activity score, ballooning, fibrosis, ALT, and inflammatory cytokines.	Among stronger probiotic trials, because histology was assessed, sample size and replication remain important.
Scorletti et al., 2020 [46]	Randomized trial in NAFLD.	Bifidobacterium animalis subsp. Lactis BB-12 plus fructo-oligosaccharides.	10–14 months	Altered fecal microbiome, but did not significantly improve liver fat or fibrosis.	An important negative trial showed that microbiome change does not necessarily translate into liver benefit.
Ayob et al., 2023 [49]	Clinical trial in NAFLD evaluating intestinal microbiota and permeability.	Multi-strain probiotics.	6 months	Explored small intestinal microbiota, cytokines, and intestinal permeability.	Mechanistically relevant, but clinical liver outcomes require cautious interpretation.
Barcelos et al., 2023 [50]	Double-blind placebo-controlled randomized study in biopsy-proven NASH.	Oral probiotic supplementation.	24 weeks	No clinically significant reduction in cardiovascular risk markers or routine metabolic/laboratory parameters.	Highlights the inconsistency and the need for patient selection and strain-specific protocols.

## Data Availability

Not applicable.

## Abbreviations

The following abbreviations are used in this manuscript:

ALT	Alanine aminotransferase
AST	Aspartate aminotransferase
CAP	Controlled attenuation parameter
CKD	Chronic kidney disease
FIB-4	Fibrosis-4 Index
LPS	Lipopolysaccharide
MAFLD	Metabolic dysfunction-associated fatty liver disease
MASLD	Metabolic dysfunction-associated steatotic liver disease
MASH	Metabolic dysfunction-associated steatohepatitis
MetALD	Metabolic alcohol-related liver disease
MRE	Magnetic resonance elastography
MRI-PDFF	Magnetic resonance imaging-proton density fat fraction
NAFLD	Non-alcoholic fatty liver disease
NASH	Non-alcoholic steatohepatitis
NFS	NAFLD Fibrosis Score
T2DM	Type 2 diabetes mellitus
VCTE	Vibration-controlled transient elastography
TNF-α	Tumor necrosis factor-alpha
IL-6	Interleukin-6
TLR4/NF-κB	Toll-like receptor 4/nuclear factor-kappa B
AMPK	AMP-activated protein kinase
ROS	Reactive oxygen species
STK39	Serine-threonine kinase 39
